# Intimate partner violence and infant morbidity: evidence of an association from a population-based study in eastern Uganda in 2003

**DOI:** 10.1186/1471-2431-7-34

**Published:** 2007-11-07

**Authors:** Charles AS Karamagi, James K Tumwine, Thorkild Tylleskar, Kristian Heggenhougen

**Affiliations:** 1 Department of Paediatrics and Child Health, Makerere University, P.O. Box 7072, Kampala, Uganda.; 2 Clinical Epidemiology Unit, Makerere University, P.O. Box 7072, Kampala, Uganda.; 3 Centre for International Health, University of Bergen, Armauer Hansen Bldg, N-5021 Bergen, Norway.; 4 Department of International Health, Boston University School of Public Health, 715 Albany Street, T4W, Boston, MA 02118, USA.

## Abstract

**Background:**

Although recent studies suggest that there is an association between intimate partner violence and child mortality, the underlying mechanisms are still unknown. It is against this background that as a secondary objective, we set out to explore whether an association exists between intimate partner violence and illness in infants.

**Methods:**

We conducted a population based household survey in Mbale, eastern Uganda in 2003. Participants were 457 women (with 457 infants) who consented to participate in the study. We measured socio-demographics of women and occurrence of intimate partner violence. We measured socio-demographics, immunization, nutritional status, and illness in the previous two weeks of the children.

**Results:**

The mean age of the women was 25 years (SD 5.7) while the mean age of the infants was 6 months (SD 3.5). The prevalence of lifetime intimate partner violence was 54% (95% CI 48%–60%). During the previous two weeks, 50% (95% CI 50%–54%) of the children had illness (fever, diarrhoea, cough and fast breathing). Lifetime intimate partner violence was associated with infant illness (OR 1.8, 95% CI 1.2–2.8) and diarrhoea (OR 2.0, 95% CI 1.2–3.4).

**Conclusion:**

Our findings suggest that infant illnesses (fever, diarrhoea, cough and fast breathing) are associated with intimate partner violence, and provide insights into previous reports that have shown an association between intimate partner violence and child mortality, suggesting possible underlying mechanisms. Our findings also highlight the importance of intimate partner violence on the health of children, and the need for further research in this area.

## Background

Intimate partner violence is the most common form of violence against women[1]. Intimate partner violence assumed international recognition initially because of its violation of women's rights. In recent years there has been accumulating evidence of the consequences of intimate partner violence on the health of women including detrimental reproductive health outcomes [2-10]. In Uganda, a hospital-based study reported that 57% of pregnant women experienced intimate partner violence [11].

However, the effect of intimate partner violence on children has received limited attention. Violence prior to or during pregnancy has been associated with premature birth, foetal injury and low birth weight[3,9,10,12]. Studies have shown that children who witness intimate partner violence are at higher risk of emotional and behavioural problems including anxiety, depression, poor school performance, low self-esteem, disobedience, nightmares and physical health complaints. Furthermore such children are likely to be perpetrators or victims of intimate partner violence as adults [13-15]. A study from India showed that women who had been beaten were significantly more likely than non-abused women to have experienced an infant death or pregnancy loss [16]. In Nicaragua, intimate partner violence was associated with an increased risk of infant and under-5 mortality [17].

Although these studies suggest that there is an association between intimate partner violence and child mortality, research on intimate partner violence and infant morbidity is lacking. Is it that intimate partner violence is associated with common childhood illnesses that eventually lead to death? As a secondary objective, we set out to explore any association between intimate partner violence and common childhood illnesses in rural and urban populations in eastern Uganda.

## Methods

### Site and design

Mbale District is situated in eastern Uganda and borders the Republic of Kenya and Mt Elgon to the east. It has a population of over 720 000 of which 90% is rural and predominantly *Bagisu *who speak *Lumasaba*. Children less than 15 years comprise 51.8 percent of the population. The literacy rate is 64 and 49 percent for males and females, respectively. The prevalence of HIV was 5.6 percent in 2003. The main economic activity is subsistence farming. Mbale District consisted of 1,448 villages distributed into 4 counties namely Bubulo, Bunghoko, Manjiya, and Mbale town. The major causes of illness in under-5 children were malaria, acute respiratory infections, measles, malnutrition and diarrhoea.

The study was a cross sectional household survey of women with infants carried out during November and December 2003. It was conducted as part of a collaborative research project between the Department of Paediatrics and Child Health, Makerere University and the Centre for International Health, University of Bergen. The field site for the research project was Mbale District. Mbale town and the surrounding Bungokho county were purposively selected because they were in the field site of the project and also to provide a rural and an urban sample. Bunghoko county was similar to the other counties of Mbale district except that it had better access to Mbale town and its services.

### Population and sampling

Sampling for the household survey was based on the WHO/EPI cluster survey method. We randomly selected 68 villages or wards (urban administrative units) each comprising of about 300 households within the county or town. Mbale town was over-sampled in order to get a good estimate also in the denser urban population. Each village or ward constituted a cluster. With the help of local officials, we identified the centre of each cluster. At the centre of the cluster, a bottle was spun on the ground and the direction in which the top of the bottle pointed was taken to be the direction of the survey. The households between the centre and boundary of the cluster were identified and the first household was randomly selected. The second household was defined as the one nearest the first household moving in the chosen direction. Subsequent households were selected in a similar manner and at the boundary of the cluster, the interviewers turned clockwise and continued to select households until a total of seven households were identified. Only households that fulfilled the selection criteria were selected. The target population was women between 15 and 45 years of age; who resided in the selected households in Mbale town or Bungokho county; and whose youngest child was aged one year or less. After consenting, the women were interviewed in their homes. In case of twins, one child was selected randomly.

### Sample size estimation

We based our sample size calculation on estimation of the prevalence of intimate partner violence in rural and urban women in Mbale. We used an expected proportion of intimate partner violence of 0.15 estimated from the study by Koenig et al [6]and the reports from neighbouring Tanzania[8]. We set the degree of precision at the standard of 0.05 and the confidence level at 95%, and incorporated a design effect of 2.0 because of the cluster design. The estimated sample size was 392 but we increased the sample size to 476 to cater for problems that might occur in recruitment. Since some women were not available for interview, and we failed to get replacements for them, the final sample size was 457 women.

### Variables and instrument

We used an interviewer-administered questionnaire that included items on socio-demographic characteristics of the woman and her husband and intimate partner violence. Data on age, education and parity was collected on a continuous scale and categorized during analysis. Women were asked about their experience of male against female intimate partner violence involving their husbands over the past 12 months and ever. Women were asked the following questions: Has your husband ever beaten you up? Why did he beat you? Has your husband beaten you up during the past year? Have you ever been threatened by a weapon or had a weapon used against you by your husband? What was the nature of the weapon? Have you ever been kicked, bitten or hit by your husband? Have you ever been raped or otherwise sexually abused by your husband?

Lifetime intimate partner violence was defined as lifetime occurrence of any form of intimate partner violence. A variable of household socio-economic status was developed by use of principal components analysis with variables on asset ownership (bicycle, radio, television, motorcycle, car/truck, land), materials of the dwelling structure (floor, wall, roof) and ownership of poultry and intimate partner animals. We used principal components analysis to divide the households into quintiles of socio-economic status, with (1) poorest and (5) least poor. We categorised quintiles 1–3 as "poorest" and 4–5 as "least poor" which suggests that the population was generally poor.

Women were also interviewed on the characteristics of the youngest child namely age, sex, method of feeding, immunization, history of diarrhoea (two or more loose stools per day), cough with fast breathing or fever in the past two weeks. Children were weighed in their underpants using a hanging Salter scale and their lengths were measured using a stadiometer. We followed guidelines established by the World Health Organization for the collection of sensitive information on intimate partner violence [18].

We recruited 12 research assistants who were fluent in English and the local language *Lumasaba *and had experience in data collection. We trained them in sampling, interview technique, measurement of child weight and height, and ethical issues, emphasising the importance of safety of the participants and interviewers, minimization of under-reporting of sensitive information particularly on intimate partner violence, and confidentiality. We conducted a pilot survey and used the lessons to revise the procedures and instrument of the survey. The survey was conducted with the support of the local officials who assisted in the sampling procedures and introduction of the survey team to household members. The research assistants worked in pairs of a female and a male, and a pair interviewed seven respondents in one cluster in one day. The lead interviewer was a female and the role of the male was mainly to ensure the privacy of the interviews and also the safety of the participants and interviewers. The interviews were conducted in the privacy of the women's homes but away from husbands, relatives, friends or local officials. The houses were generally close together and interviews were conducted daily between 8 am and 6 pm. The research assistants were in direct communication by mobile telephone with the principal investigator (CASK) who could reach them in less than an hour.

### Data analysis

Data were entered into EPIDATA and then exported to Stata version 8.0 for analysis that adjusted for the design effect. Data quality was ensured through careful selection and training of the research assistants, supervision, field editing, and by use of the check programme at data entry. We combined the common childhood illnesses (fever, cough, fast breathing, diarrhoea) into one variable called ILL. The variable ILL was dichotomised into not/mildly ILL consisting of 0 or 1 symptom or moderately ILL consisting of 2 to 4 symptoms. This level of categorization was chosen because it had the best combination of sensitivity (57%) and specificity (59%) with intimate partner violence as outcome. The dependent variables were ILL, FEVER, COUGH WITH FAST BREATHING, DIARRHOEA, EXCLUSIVE BREASTFEEDING, COMPLETE VACCINATION, UNDERWEIGHT, WASTING, and STUNTING. A separate analysis was performed for each dependent variable against the independent variables that included the characteristics of the women, their husbands and infants. A bivariate analysis was performed between each dependent variable and the independent variables so as to determine the independent variables that were associated with each dependent variable. The independent variables that were significantly associated with each dependent variable were potential confounders and were entered into a model for logistic regression. When ILL was used as the dependent variable, its component variables (fever, cough, fast breathing, and diarrhoea) were not included in the analysis as independent variables so as to avoid interaction. Odds ratios were used to estimate the strength of the associations while 95% confidence intervals were used for significance testing.

### Ethics

Ethical and institutional approval for the study was obtained from Makerere University Faculty of Medicine Research and Ethics Committee and informed consent was obtained from all participants in the study.

## Results

### Characteristics of women

Our sample consisted of 457 women and infants (Table 1). Over half the women were rural (58%), with a mean age of 25 years (SD 5.7), Muslim (62%), and married (91%). The majority of the women were multiparous (78%), had less than 8 years of education (71%), and worked in agriculture (88%). Most women had attended an antenatal clinic during the most recent pregnancy (97%) but only 47 percent delivered in a health unit. The prevalence of lifetime intimate partner violence was 54 percent.

**Table 1:** Characteristics of women, husbands and infants in Mbale, Uganda, 2003.

**Variable**	**Frequency**	**Percentage**
Age of mother (years)		
15 – 24	238	52
25 – 45	219	48
Education of mother (years)		
0 – 7	324	71
8 or more	133	29
Marital status		
Single/widow	191	46
Married	227	54
Parity		
Primipara	100	22
Multipara	357	78
Mother works outside the home		
No	123	27
Yes	334	73
Residence		
Urban	124	27
Rural	333	73
Socio-economic status		
Least poor	122	40
Poorest	185	60
Lifetime intimate partner violence		
No	208	46
Yes	249	54
Age of husband (years)		
15 – 24	69	21
25 – 45	260	79
Education of husband (years)		
0 – 7	212	57
8 or more	160	43
Number of people in household		
5 or less	220	48
6 or more	237	52
Age of child (months)		
<6	220	48
6–12	237	52
Sex of child		
Male	255	56
Female	202	44
Is child on exclusive breastfeeding?		
Yes	105	23
No	348	77
Complete vaccination for age		
No	252	55
Yes	205	45
Is child wasted?		
No	427	93
Yes	30	7
Is child underweight?		
No	407	89
Yes	50	11
Is child stunted?		
No	421	92
Yes	36	8
Has child had cough with fast breathing in past two weeks?		
No	413	90
Yes	44	10
Has child had fever in past two weeks?		
No	225	49
Yes	232	51
Has child had diarrhoea in past two weeks?		
No	322	70
Yes	135	30

### Characteristics of children

There were 457 infants with a mean age of 6 months (SD 3.5) and a range of 0 to 12 months (Table 1). The male to female ratio was 1.3 to 1. Twenty three percent of the infants were exclusively breastfed over the past 24 hours while 45 percent were fully vaccinated for age. The prevalence of wasting was 7 percent, underweight was 11 percent, and stunting was 8 percent. During the previous two weeks, 30 percent of the infants had experienced diarrhoea, 10 percent had cough with fast breathing and 51 percent had fever.

### Association between lifetime intimate partner violence and illness, exclusive breastfeeding, and nutritional status of infants

Lifetime intimate partner violence was significantly associated with infant illness in both the unadjusted (OR 1.9, 95% CI 1.3–2.7) and adjusted analyses (OR 1.8, 95% CI 1.2–2.8) (Tables 2 and 3). Furthermore, lifetime intimate partner violence was associated with diarrhoea, fever and cough with fast breathing, though only diarrhoea (OR 2.0, 95% CI 1.2–3.4) achieved significance (Table 4). Lifetime intimate partner violence was associated with education of mother, parity and exclusive breastfeeding in the unadjusted analysis but not in the logistic regression (Tables 3 and 4).

**Table 2:** Bivariate association between illness in infants and characteristics of women, husbands and infants, Mbale, Uganda, 2003.

**Variable**	**Illness n (%)**	**No Illness n (%)**	**Unadjusted OR (95% CI)**
Age of mother (years)			
15 – 24	122 (51)	116 (49)	1.1 (0.8–1.6)
25 – 45	106 (48)	113 (52)	1.0
Education of mother (years)			
0 – 7	177 (55)	147 (45)	**1.9 (1.3–2.9)**
8 or more	51 (38)	82 (62)	1.0
Marital status			
Single/widow	91 (48)	100 (52)	1.0
Married	121 (53)	106 (47)	1.3 (0.9–1.8)
Parity			
Primipara	39 (39)	61 (61)	1.0
Multipara	189 (53)	168 (47)	**1.8 (1.1–2.8)**
Mother works outside the home			
No	61 (50)	62 (50)	1.0
Yes	167 (50)	167 (50)	1.0 (0.7.1.5)
Residence			
Urban	42 (34)	82 (66)	1.0
Rural	186 (56)	147 (44)	**2.5 (1.6–3.8)**
Socio-economic status			
Least poor	63 (52)	59 (48)	1.0
Poorest	100 (54)	85 (46)	1.1 (0.7–1.7)
Age of husband (years)			
15 – 24	39 (57)	30 (43)	1.5 (0.9–2.5)
25 – 45	121 (47)	139 (53)	1.0
Education of husband (years)			
0 – 7	116 (55)	96 (45)	1.4 (0.9–2.1)
8 or more	74 (46)	86 (54)	1.0
Number of people in household			
5 or less	101 (46)	119 (54)	1.0
6 or more	109 (46)	128 (54)	1.0 (0.7–1.5)
Age of child (months)			
<6	81 (37)	139 (63)	1.0
6–12	147 (62)	90 (38)	**2.8 (1.9–4.1)**
Sex of child			
Male	121 (47)	134 (53)	1.0
Female	107 (53)	95 (47)	1.2 (0.9–1.8)
Is child on exclusive breastfeeding?			
Yes	36 (34)	69 (66)	1.0
No	189 (54)	159 (46)	**2.3 (1.4–3.6)**
Complete vaccination for age			
No	139 (55)	113 (45)	1.0
Yes	89 (43)	116 (57)	0.6 (0.4–0.9)
Is child wasted?			
No	211 (49)	216 (51)	1.0
Yes	17 (57)	13 (43)	1.3 (0.6–2.8)
Is child underweight?			
No	205 (50)	202 (50)	1.0
Yes	23 (46)	27 (54)	0.8 (0.5–1.5)
Is child stunted?			
No	210 (50)	211 (50)	1.0
Yes	18 (50)	18 (50)	1.0 (0.5–2.0)
Lifetime intimate partner violence			
No	86 (41)	122 (59)	1.0
Yes	142 (57)	107 (43)	**1.9 (1.3–2.7)**

**Table 3:** Logistic regression of illness in infants against characteristics of women, husbands and infants, Mbale, Uganda, 2003.

**Variable**	**Illness n (%)**	**No Illness n (%)**	Adjusted* OR (95% CI)
Education of mother (years)			
0 – 7	177 (55)	147 (45)	0.7 (0.4–1.1)
8 or more	51 (38)	82 (62)	1.0
Parity			
Primipara	39 (39)	61 (61)	1.0
Multipara	189 (53)	168 (47)	1.5 (0.9–2.6)
Residence			
Urban	42 (34)	82 (66)	1.0
Rural	186 (56)	147 (44)	**2.0 (1.2–3.3)**
Age of child (months)			
<6	81 (37)	139 (63)	1.0
6–12	147 (62)	90 (38)	**2.9 (1.8–4.4)**
Is child on exclusive breastfeeding?			
Yes	36 (34)	69 (66)	1.0
No	189 (54)	159 (46)	1.4 (0.8–2.3)
Lifetime intimate partner violence			
No	86 (41)	122 (59)	1.0
Yes	142 (57)	107 (43)	**1.8 (1.2–2.8)**

* Association between lifetime intimate partner violence and illness in infants adjusted for education of mother, parity, residence, age of child, and exclusive breastfeeding.

**Table 4:** Summary of the associations between intimate partner violence, illness and nutritional status of infants, Mbale, Uganda, 2003.

**Variable**	**Violence n (%)**	**No Violence n (%)**	**Unadjusted OR (95% CI)**	Adjusted* OR (95% CI)
Is child on exclusive breastfeeding?				
Yes	55 (52)	50 (48)	1.0	1.0
No	193 (55)	155 (45)	1.1 (0.7–1.8)	0.8 (0.4–1.5
Complete vaccination for age				
No	139 (55)	113 (45)	1.0	1.0
Yes	110 (54)	95 (46)	0.9 (0.7–1.4)	1.0 (0.7–1.5)
Is child wasted?				
No	236 (55)	191 (44)	1.0	1.0
Yes	13 (43)	17 (57)	0.6 (0.3–1.3)	0.7 (0.3–1.7)
Is child underweight?				
No	229 (56)	178 (43)	1.0	1.0
Yes	20 (40)	30 (60)	**0.5 (0.3–0.9)**	0.7 (0.3–1.5)
Is child stunted?				
No	232 (55)	189 (45)	1.0	1.0
Yes	17 (47)	19 (53)	0.7 (0.4–1.4)	0.9 (0.3–3.3)
Has child had cough with fast breathing in past two weeks?				
No	218 (53)	195 (47)	1.0	1.0
Yes	31 (70)	13 (30)	**2.1 (1.1–4.2)**	1.7 (0.9–3.5)
Has child had fever in past two weeks?				
No	109 (48)	116 (52)	1.0	1.0
Yes	140 (60)	92 (40)	**1.6 (1.1–2.3)**	1.3 (0.8–1.9)
Has child had diarrhoea in past two weeks?				
No	161 (50)	161 (50)	1.0	1.0
Yes	88 (65)	47 (35)	**1.9 (1.2–2.8)**	**2.0 (1.2–3.4)**

Note:

Intimate partner violence was the major exposure variable.

The child illness and nutrition variables were the dependent variables.

* Odds ratios were adjusted for education of mother, parity, residence, and age of child.

## Discussion

Infants of women who reported intimate partner violence were twice as likely to have had an acute illness (diarrhoea, fever, cough and fast breathing) in the past two weeks compared to infants of women who did not report intimate partner violence. Furthermore, diarrhoea, fever and cough with fast breathing were individually associated with lifetime intimate partner violence although only diarrhoea achieved significance probably because of a higher frequency. To our knowledge, this is the first time that infant illness has been associated with intimate partner violence. The association between infant illness and intimate partner violence could be due to the potential effect of intimate partner violence on breastfeeding. Intimate partner violence involves physical, emotional and sexual coercion of women [1]. This coercion is likely to create stress, and in a breastfeeding woman, stress inhibits the release of oxytocin, the hormone responsible for the letdown reflex that facilitates the flow of breast milk[19]. The stress of intimate partner violence may thus lead to inadequate breast milk and the commonly reported problem of not enough milk[20,21]. Infant illness would result from lack of the protective effect of breastfeeding combined with exposure to other foods that may be contaminated or cause allergy.

It is also plausible that infant illness may be the trigger to intimate partner violence. Women are usually blamed for all kinds of misfortune including illness in the child[22]. Thus the husband may blame his wife when the infant is ill and this situation may explode into intimate partner violence. More likely however, the husband, particularly in a poor family and out of frustration, may respond with violence if his wife asks him for money that he does not have in order to take the child for treatment. A third explanation for the association between illness in infants and intimate partner violence is that both conditions may be the consequences of similar underlying factors. Our sample consisted of mainly the poorest and least educated women. Both illness in infants [23,24] and intimate partner violence[15,22,25] have been associated with poverty and low female education.

Consistent with other studies, our study showed that illness was more frequent in older infants [23,24,26,27]. Older infants are usually on a combination of breastfeeding and locally available foods [28]. In our study, almost all the children aged 6 to12 months were on a combination of breastfeeding and other foods. In such a situation, the protective effect of breastfeeding is decreased while introduction of other foods further increases the risk of infection[19,27]. In addition, the child developmental stages including sitting, crawling and putting objects into the mouth increasingly expose the infants to environmental pathogens and thereby increase the risk of illness[19].

Infants residing in rural areas had a three-fold increase in common illnesses. Our study showed that more than 80% of the less educated mothers resided in rural areas. The education of the mother is strongly associated with childhood illness, and low or no education has been shown to increase the risk of childhood illness [23]. The possible mechanisms for the increased risk of childhood illness among uneducated mothers include inappropriate infant feeding practices such as mixed breastfeeding, and poor hygiene [23]. Other factors such as poverty and environmental hygiene may also have played a role in increasing the risk of childhood illness among rural women [24].

In the unadjusted analysis, our study showed that low or no education, multiparity, and non-exclusive breastfeeding were associated with an increased risk of illness in infants. However, this association was lost in the adjusted analysis. This is contrary to previous studies that have demonstrated an association between maternal education, multiparity and non exclusive breastfeeding with illness in children [29-31]. The lack of significant association may be due to inadequate power in our study to examine the relationships and possibly to exclusion of non-significant but important socio-demographic variables from the logistic regression model.

Our study had a number of potential limitations. The study was cross sectional and it was therefore not possible to establish a causal association between intimate partner violence and infant illness. In addition, the study could not establish a significant association between intimate partner violence and fever or cough and fast breathing because of a limited sample size. A further potential limitation of the study was the seasonal variation of childhood illnesses and it is uncertain whether our findings would still be valid over a longer period of study. A final potential limitation of our study was the measurement of intimate partner violence. The cultural definition of intimate partner violence, particularly sexual coercion is ambiguous. Furthermore, intimate partner violence is a sensitive issue that is often hidden by the women. Finally, intimate partner violence was measured as a lifetime experience whereas infant illness was measured over the past two weeks. These factors may either singly or in combination have introduced bias in our study.

## Conclusion

Notwithstanding these limitations, our findings suggest that infant illnesses (fever, diarrhoea, cough and fast breathing) are associated with intimate partner violence, and provide insights into previous reports that have shown an association between intimate partner violence and child mortality, suggesting possible underlying mechanisms. Our findings also highlight the importance of intimate partner violence on the health of children, and the need for further research in this area.

## Competing interests

The author(s) declare that they have no competing interests.

## Authors' contributions

CASK participated in the conception, design, and implementation of the study, statistical analysis, interpretation and drafting of manuscript. JKT participated in study conception, design and implementation of the study. TT participated in study conception, design, statistical analysis, and interpretation. KH participated in study conception, design, and interpretation. All authors read and approved the final manuscript.

## Pre-publication history

The pre-publication history for this paper can be accessed here:


